# A family history: effects of parental age on offspring life-history traits in bighorn sheep

**DOI:** 10.1093/beheco/araf046

**Published:** 2025-05-08

**Authors:** Emmanuelle Marchand, Limoilou-Amélie Renaud, Marco Festa-Bianchet

**Affiliations:** Département de Biologie, Université de Sherbrooke, 2500, boul. de l’Université, Sherbrooke, Québec, J1K 2R1, Canada; École d'études autochtones, Université du Québec en Abitibi-Témiscamingue, QC, J9X 5E4, Canada; Département de Biologie, Université de Sherbrooke, 2500, boul. de l’Université, Sherbrooke, Québec, J1K 2R1, Canada

**Keywords:** Lansing effect, life history, longevity, long-term study, *Ovis canadensis*, parental age

## Abstract

Long-term fitness effects of parental age could affect population dynamics, as age structure can vary considerably over time. Few studies of wild animals, however, have examined the possible long-term fitness consequences of parental age. Based upon 50 years of data on wild bighorn sheep, we investigated how parental age affected three offspring traits—mass at three years, longevity, and female lifetime reproductive success. We also tested for a survival filter which could mask or increase fitness effects of parental age. Our results showed a significant, quadratic negative association between maternal age, offspring longevity and female lifetime reproductive success. Offspring born to mothers aged 5 to 6 yr lived about 2 yr longer and weaned about 1.5 lambs more than siblings born to mothers aged 12 to 13 yr. The effect of paternal age was not significant. There was also a positive association between the longevity of mothers and offspring. We did not detect any effects of parental age on mass at three years for offspring of either sex. These results demonstrate the presence of persistent maternal age effects in a long-lived species. The sex-specific effects support the importance of analyzing maternal and paternal age effects separately, as well as effects on female and male offspring. This study advances our understanding of evolutionary processes and population dynamics in wild long-lived mammals.

## Teaser

In bighorn sheep, offspring of older mothers had shorter life expectancy and lifetime reproductive success than their siblings born when the mother was younger. There was no effect of paternal age on offspring traits. The age of mothers and fathers did not affect offspring adult mass. Age structure of mothers could affect population dynamics over the long run: if most mothers are old, subsequent life expectancy and reproductive success will be reduced.

## Introduction

In the early 20^th^ century, Alexander Graham Bell (1918) found that children born to younger parents outlived their counterparts born to older parents by nearly a decade. Building upon this foundation, Albert Lansing (1947) work on rotifers revealed a striking pattern—older breeding lines tended to produce smaller offspring with shorter lifespan compared to younger parental lines. Lansing postulated the existence of a “transmissible and cumulative aging factor,” which was more pronounced in the offspring of older parents. This pattern is now widely recognized as the “Lansing effect.”

Since then, most studies on the subject have been conducted in laboratories, and few concerned long-lived species (Monaghan et al. 2020). Identifying parentage age effects in wild populations is difficult, as it requires a pedigree with known-age parents and a longitudinal dataset of offspring and parents monitored over their lifetime. Consequently, there is little information on paternal age effects that may manifest after the end of parental care in natural populations. Captivity-based research offers some evidence of parental age influencing offspring fitness (Sharman et al. 2022). The age of both parents can have negative effects on offspring fitness. Studies of semi-captive Asian elephants (*Elephas maximus*) noted that offspring born to older mothers exhibit lower overall survival rates but higher reproductive success, alongside a decrease in survival rates among their own progeny (Reichert et al. 2020). An influence of paternal age has also been detected, even when paternal care is absent. For instance, in houbara bustard (*Chlamydotis undulata*) experimental insemination by older fathers tends to produce chicks with slower growth rates (Preston et al. 2015).

Given that the age structure of populations can vary considerably over time (Festa-Bianchet et al. 2003), long-term fitness effects related to parental age could affect population dynamics (Benton et al. 2008). Thus, the investigation of whether parental age influences independent offspring is crucial to improve our understanding of drivers of population growth rate within natural populations and, in turn, enhance management measures.

The cross-sectional design that has been most commonly used to investigate the effects of parental age (Monaghan et al. 2020) does not account for individual variations. Notably, it assumes that differences in parental abilities are independent of survival to different ages. This limitation might lead to an overestimation of parental abilities of older individuals if less capable parents had lower survival rates, leading to selective disappearance of parents with low reproductive potential earlier in life. The presence of a relation between survival and parental phenotype could mask or increase the effects of parental age on offspring fitness if not considered (van de Pol and Verhulst 2006). To properly quantify any effects of parental age, longitudinal studies tracking individuals of known age through their lifespan are required.

We exploited a long-term dataset of intensively monitored wild bighorn sheep (*Ovis canadensis*) to investigate whether parental age influenced offspring fitness through three life-history traits—mass at three years, longevity, and female lifetime reproductive success. We also tested an effect of selective disappearance of parents. Based on the Lansing effect, we predicted a decline in offspring fitness as parents age. Given that females continue to grow from 2 to 4 yr of age (Festa-Bianchet et al. 1996), and survival senescence starts at around 7 yr (Loison et al. 1999), we expected a bell-shaped effect of maternal age on offspring traits, with an initial improvement followed by a decline. Due to the absence of paternal care in this species, any effects of paternal age are likely attributed solely to the transfer of sperm and genes. Therefore, we did not expect to observe any changes in offspring fitness prior to paternal senescence. If a survival filter existed, wherein “good” parents were more likely to survive to an older age, we anticipated a positive effect of parental age unless differences in parental longevity were accounted for.

Bighorn sheep show senescence in survival beginning at about 7 yr of age (Loison et al. 1999). Heavier females have longer life expectancy, but show a decrease in mass from about 11 yr of age (Bérubé et al. 1999). Maternal age effects on lambs have been previously investigated (Jorgenson et al. 1997; Festa-Bianchet and King 2007) and reveal a later onset of reproductive senescence, beginning at about 13 yr, an age not reached by most females because of survival senescence. There have been no investigations of possible effects of parental age later in life, and similarly to most mammals, the effects, if any, of paternal age are unknown. We sought to examine whether increasing parental age had positive effects on adult offspring traits, as expected if experience and possible selective survival improved apparent parental performance with age, or negative effects as expected if parental senescence played a greater role. To focus on long-term effects, we only considered progeny that survived to one year of age and older.

## Materials and methods

### Study area and population

The bighorn sheep population of Ram Mountain, Alberta, Canada (52° N, 115° W), has been monitored since 1971 (Festa-Bianchet et al. 2019). Ram Mountain is approximately 30 km east of the main Rocky Mountains range. It is separated from the Rocky Mountains by coniferous forest and from Shunda Mountain by the North Saskatchewan River, creating an isolated study area covering around 38 km^2^ of alpine tundra and subalpine meadows. Monitoring of bighorn sheep is based upon a capture-mark-recapture method and sheep are captured using a corral trap. The trap is baited with salt from late May to late September. Between 1975 and 2002, we caught 934 lambs. Of 111 sheep not caught as lambs during this period, 79 (71%) were caught as yearlings. Upon the first capture of each sheep, typically as lamb or yearling, sex is determined and individuals are marked with either an ear tag or a visual collar, allowing for distant identification. Capture frequency is one to nine times per summer and around 85% of sheep are caught at least once each summer (Martin and Pelletier 2011). Mass is measured at each capture to the nearest 250 g with a Detecto spring scale, then adjusted to June 5 and September 15, corresponding to the approximate beginning and end of the vegetation growing season, using a GLMM as detailed in (Martin and Pelletier 2011). Population size was measured each year in June as the number of adult females aged two years and older. Population size is a good proxy of density because females use the same area year after year. Resighting probability is > 99% for females (Festa-Bianchet et al. 2003) and > 95% for males (Jorgenson et al. 1997). The age of all individuals included in analyses is known, and longevity can be confidently assigned because of the very high resighting rate. Maternal age is the age at parturition, while paternal age is the age at siring. Sheep that were artificially removed (Jorgenson et al. 1993), killed by hunters, died due to capture incidents or emigrated were not considered.

### Pedigree—maternity and paternity assignment

Since 1971, maternity has been determined through the observation of suckling behavior. Paternities became ascertainable from 1988, when DNA analyses of samples collected during capture were started. Paternity assignments were based on approximately 30 microsatellite loci and utilized the 95% confidence threshold in CERVUS software (see Coltman et al. (2005) and Marshall et al. (1998) for details). Progeny sired by immigrant males of unknown identity or age, that are only present for the rut (Pigeon et al. 2016; Ritchot et al. 2021), were excluded from subsequent analyses involving paternal age. The pedigree available for this study included 973 maternal links from 284 ewes (mean number of offspring ± SD = 3.43 ± 2.52) and 401 paternal links from 93 rams (offspring per sire = 4.31 ± 4.62).

### Offspring traits studied

Mass at three years of age: For both sexes, body mass was adjusted to September 15 (Figure S1) (Martin and Pelletier 2011). Associations between mass of the same individual on September 15 in consecutive years are stronger than similar associations for mass adjusted to June 5, and mass in mid-September represents the seasonal peak in mass accumulation (Festa-Bianchet et al. 1996). Both sexes are sexually mature at this age. Females have achieved about 90% of their asymptotic growth, and heavier young females have a greater life expectancy (Festa-Bianchet et al. 1996; Bérubé et al. 1999). Therefore, female body mass at age 3 reflects adult mass. Males have achieved about 75% of their asymptotic mass at three years of age (Festa-Bianchet et al. 1996) but because of high male mortality (Loison et al. 1999), we selected this age to ensure a sufficient sample size.

Longevity: For both sexes, age at death. Because of the very high resighting probability, animals were assumed to have died in the last year they were seen.

Lifetime reproductive success (LRS): For females only, the number of lambs produced (reported in Supplementary) or weaned. Females that were older than three years at the start of the study (n = 38), received a contraceptive implant (n = 12), died before two years of age and could not have reproduced (n = 47), or were alive and younger than nine years in 2024 (n = 12) were excluded. We included four ewes alive in 2024 and aged 12 to 18 yr, near the end of their reproductive lifespan. We did not estimate lifetime reproductive success for males because not all lambs were sampled for DNA (Van de Walle et al. 2022), and some males leave for the rut (Lassis et al. 2022).

### Statistical analyses

We modeled the influence of parental age on mass at three years, longevity and female LRS by explicitly incorporating within-individual changes (*β*_*W*_, senescence) and between-individual differences (*β*_*S*_, selective disappearance) following the approach of van de Pol and Verhulst (2006). Maternal and paternal ages could not be included in the same models because fewer fathers than mothers were known. Therefore, we conducted separate analyses testing the effects of maternal and paternal age on reproductive traits.

To account for covariation at different levels of aggregation (within vs. between individual) and ensure unbiased estimation of age effects, we used generalized linear mixed models (GLMMs) with both parental age and longevity included as fixed effects. Under this framework, senescence refers to a decline in reproductive performance within individuals as they age, whereas selective disappearance captures the correlation between an individual’s longevity and its underlying ‘quality’. By including both parental age and longevity as fixed effects, we explicitly tested for within-individual changes in reproductive performance across years (*β*_*W*_ × age_ij_) while accounting for selective disappearances (*β*_*S*_ ×ω_i_) and vice versa, using a two-level model where individual identity (_*i*_) was the highest level, and the annual measurement of reproductive traits (_*j*_) was the lowest level. Parental longevity is also a proxy of individual reproductive potential, as females are limited to one lamb per year and longevity is correlated with adult mass (Bérubé et al. 1999).

Mass at three years was modeled using a Gaussian distribution, longevity using a binomial distribution, and female LRS using a zero-inflated Poisson distribution (Figure S1). All models were implemented with the “glmmTMB” package (Brooks et al. 2017). We assessed model fit by examining diagnostic plots of residuals and tested for overdispersion using the “DHARMa” package (Hartig 2024). Both linear and quadratic terms were tested for maternal age. We included covariates known to influence offspring traits: population density at three years (models of mass) or at birth (models of longevity, female LRS) (Leblanc et al. 2001; Pigeon et al. 2017), sex (Festa-Bianchet et al. 1994, 2000; Jorgenson et al. 1997), and the interaction between offspring sex and parental age. Covariates were mean-centered and scaled by their standard deviation to facilitate model convergence. Only significant covariates were retained after selection using a backward stepwise procedure to ensure model parsimony.

To account for repeated measures and genetic relationships, we first tested whether including maternal or paternal identity as random effects improved model fit, thus accounting for possible non-independence of siblings. We assessed the significance of random effects using likelihood ratio test (LRTs), which compare the fit of nested models by testing whether the removal of a random effect significantly reduces model likelihood. The LRT follows a chi-square distribution, and a random effect was considered significant if its removal led to a significant decrease in model fit (*P* < 0.05). Additionally, we assessed the inclusion of offspring birth (models of mass at three years) and death years (offspring longevity and female LRS models) as random effects to capture cohort effects and environmental variations in forage quality, weather conditions, and periods of intense cougar (*Puma concolor*) predation (Cloutier et al. 2024). We tested the significance of these effects using LRTs.

Because we were interested in parental age effects on fitness after weaning, we limited analyses to offspring aged one year and older to avoid possible direct effects of lactation (Wilson et al. 2005). Longevity analyses included offspring from cohorts born between 1971 and 2013. Of these, all but four individuals were deceased, ensuring minimal bias due to right censoring.

Results are presented as estimates (*β*) with 95% confidence intervals (CIs, [lower limit, upper limit]). Effects were considered significant when CIs did not overlap zero. All analyses were conducted in R version 4.4.2 (R Core Team, 2024). 

## Results

From 1971 to 2024, we estimated effects of parental age on three life-history traits: mass at three years, female LRS and longevity. Sample sizes for each analysis varied because not all variables were available for all offspring. Maternal age at birth ranged from 2 to 17 years (mean = 6.6; median = 6.0; n = 284) and paternal age at conception from 2 to 14 years (mean = 6.4; median = 6.0; n = 93). Maternal longevity ranged from 2 to 19 yr (mean = 10.4; median = 10.0; n = 284) and paternal longevity from 2 to 14 yr (mean = 8.6; median = 9.0; n = 93). Population density varied from 16 to 103 adult females in June (mean = 52.8; median = 47.0). Table S1 reports range, mean and median for parental age and longevity and for population density. The Pearson correlation between maternal and paternal age was 0.10 [95% CI: 0.006, 0.20], indicating a significant but weak association (Figure S2). In females, offspring longevity and lifetime reproductive success were strongly correlated (r = 0.86). In contrast, longevity and mass at 3 yr showed only weak correlations in both female (r = 0.02) and male offspring (r = -0.16).

### Offspring mass at three years

We analyzed the effects of maternal and paternal age on offspring mass at three years, with separate models for each parent. For maternal age effects, we included within-mother variation in age, and between-mothers effects (maternal age at last sighting, our proxy of longevity) along with offspring sex and female density when the offspring was three years old. We used a similar approach for paternal age effects.

#### Maternal age:

We found no evidence that maternal age affected offspring mass at three years, either within-mother (age) or between-mothers (longevity) (Table 1, Figure S3). The quadratic term for maternal age (*β =* -0.40 [-0.99, 0.18]) and the interaction between maternal age and offspring sex (*β =* 0.33 [-1.13, 1.78]) were not significant and were dropped from the model. Only maternal identity and birth year were retained as random intercepts, as paternal identity did not significantly improve model fit (LRT: *χ*^2^_1_ = 2.55, p = 0.11), and death year was not relevant for this trait.

**Table 1.** Estimates of fixed effects and standard deviations of random intercepts, along with their corresponding 95% confidence intervals (95% CI), from models examining the relationship between offspring life-history traits and maternal age in bighorn sheep at Ram Mountain, Alberta, Canada (1971 – 2024). Offspring mass was modeled using a Gaussian distribution, longevity using a negative binomial distribution, and female lifetime reproductive success using a zero-inflated Poisson distribution. Estimates for longevity and lifetime reproductive success are presented on the log scale.

	Offspring mass at three years 1971 – 2024 N = 221 offspring, 112 mothers		Offspring longevity Cohorts 1971 – 2013 N = 290 offspring, 124 mothers		Female offspring lifetime reproductive success 1971 – 2024 N = 146 offspring, 95 mothers	
Parameter	Estimate	95% CI	Estimate	95% CI	Estimate	95% CI
Intercept	63.85	62.69, 65.01	1.91	1.74, 2.07	1.36	1.14, 1.55
Maternal age	0.15	-0.62, 0.93	-0.14	-0.21, -0.06	-0.14	-0.30, 0.02
Maternal age^2^	–	–	-0.06	-0.12, -0.01	-0.14	-0.27, -0.02
Maternal longevity	0.40	-0.49, 1.29	0.09	0.01, 0.18	0.17	0.03, 0.32
Offspring sex (level: male)	18.26	16.82, 19.7	-0.19	-0.31, -0.08	–	–
Female density	-1.36	-2.42, -0.31	-0.14	-0.23, -0.05	-0.10	-0.27, 0.06
*Variance (standard deviation) and model performance*						
Maternal identity	2.43	1.56, 3.79	0.32	0.25, 0.43	0.33	0.20, 0.56
Birth year	1.78	1.03, 3.07	–	–	–	–
Death year	–	–	0.34	0.25, 0.47	0.21	0.07, 0.61
Conditional *R*^*2*^	0.81	–	0.67	–	0.23	–
Marginal *R*^*2*^	0.73	–	0.14	–	0.09	–

#### Paternal age:

We found no significant effects of paternal age on offspring mass at three years (Table 2, Figure S3). The interaction between paternal age and offspring sex (*β =* 0.43 [-1.83, 2.69]) and the quadratic term for age (*β* = -0.19 [-1.04, 0.78]) were excluded from the final model. We included paternal identity and birth year as random effects in the final model, as maternal identity did not significantly improve model fit (LRT: *χ*^2^_1_= 0.34 p = 0.56). We observed strong sexual dimorphism in mass at three years (Figure S2) and a negative effect of population density on offspring mass (Tables 1 and 2).

**Table 2.** Estimates of fixed effects and standard deviations of random intercepts, along with their corresponding 95% confidence intervals (95% CI), from models examining the relationship between offspring life-history traits and paternal age in bighorn sheep at Ram Mountain, Alberta, Canada (1988 – 2024). Offspring mass was modeled using a Gaussian distribution, longevity using a negative binomial distribution, and female lifetime reproductive success using a zero-inflated Poisson distribution. Estimates for longevity and lifetime reproductive success are presented on the log scale.

	Offspring mass at three years 1988 – 2024 N = 98 offspring, 42 fathers		Offspring longevity Cohorts 1988 – 2013 N = 120 offspring, 42 fathers		Female offspring lifetime reproductive success 1988 – 2024 N = 155 offspring, 28 fathers	
Parameter	Estimate	95% CI	Estimate	95% CI	Estimate	95% CI
Intercept	63.33	61.7, 64.96	1.73	1.47, 1.94	0.88	0.48, 1.25
Paternal age	0.52	-1.15, 2.19	-0.05	-0.21, 0.11	0.30	-0.11, 0.73
Paternal age^2^	–	–	–	–	–	–
Paternal longevity	-1.69	-3.45, 0.07	-0.14	-0.3, 0.01	-0.46	-0.95, 0.01
						
Offspring sex (level: male)	18.61	16.39, 20.83	-0.09	-0.34, 0.14	–	–
Female density	-2.14	-3.67, -0.61	0.12	-0.05, 0.32	0.03	-0.39, 0.46
*Variance (standard deviation) and model performance*						
Paternal identity	1.35	0.23, 8.04	0.15	0.02, 0.93	0.27	0.01, 0.75
Birth year	1.89	0.87, 4.11	–	–	–	–
Death year	–	–	0.39	0.22, 0.70	0.29	0.01, 0.76
Conditional *R*^*2*^	0.80	–	0.54	–	0.27	–
Marginal *R*^*2*^	0.75	–	0.13	–	0.12	–

### Offspring longevity

#### Maternal age:

**Fig. 1.**
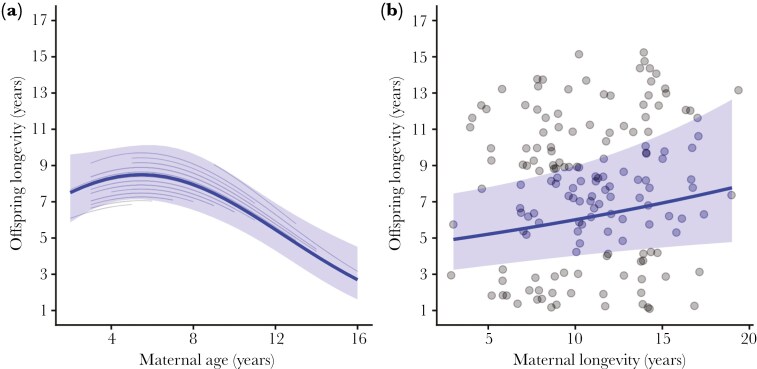
Offspring longevity for bighorn sheep at Ram Mountain, Alberta, Canada (1971-2013). Solid lines represent model predictions for female offspring under average female density conditions (results for male offspring are reported in Table 1). Shaded bands represent 95% confidence intervals while gray lines are modeled trends for individual mothers. (A) Offspring longevity as a function of maternal age at birth. (B) Offspring longevity as a function of maternal longevity, estimated by the age at last sighting.

The quadratic effect of maternal age on offspring longevity was significant and negative (*β*_*age (linear)*_*=* -0.14 [-0.21, -0.06]; *β*_*age (quadratic)*_* = *-0.06 [-0.12, -0.01], Fig. 1, Table 1).

Given the log link function, this result suggests that the rate of decline in offspring longevity increases as maternal age increases. Exponentiating the coefficient (exp (-0.06) ≈ 0.94) suggests that for every unit increase in maternal age, expected offspring longevity decreased by approximately 6%, with the rate of decline increasing at older maternal ages. When we examined a reduced model excluding maternal longevity, we found similar but weaker estimates for maternal age (*β*_*age (linear)* =_ -0.12 [-0.20, -0.05]; *β*_*age (quadratic)*_ = -0.06 [-0.12, 0.00]). Maternal longevity did not appear to substantially affect the estimated relationship between maternal age and offspring longevity, and the reduced model provided a comparable fit to the data. Offspring sex, as an additive effect, was significant, suggesting that maternal age had a significant negative effect on longevity for both female and male offspring, with male offspring having shorter lifespan that female offspring overall (*β*_*sex(male)*_ = -0.19 [-0.31, -0.08]). The effect of maternal age did not differ between male and female offspring, as the interaction between maternal age and offspring sex was not significant and was dropped from the model. Female density in the year of birth, included as a control variable, was significant (*β* = -0.14 [-0.23, -0.05]). Maternal longevity was positive and significant (*β* = 0.09 [0.01, 0.18], suggesting that maternal longevity explained offspring lifespan even after controlling for within-mother changes in maternal age. Maternal identity and death year were both included as random intercepts and had nonzero effects on longevity (Table 1).

#### Paternal age:

Paternal age had no significant effects on offspring longevity (*β* = -0.05 [-0.21, 0.11], Table 2, Figure S4 ). We found no effect of father longevity on offspring longevity (-0.14 [-0.3, 0.01]), and no effect of population density or offspring sex (Table 2). Small amounts of variance were explained by father identity and death year as random intercepts (Table 2).

### Female offspring lifetime reproductive success

#### Maternal age:

**Fig. 2.**
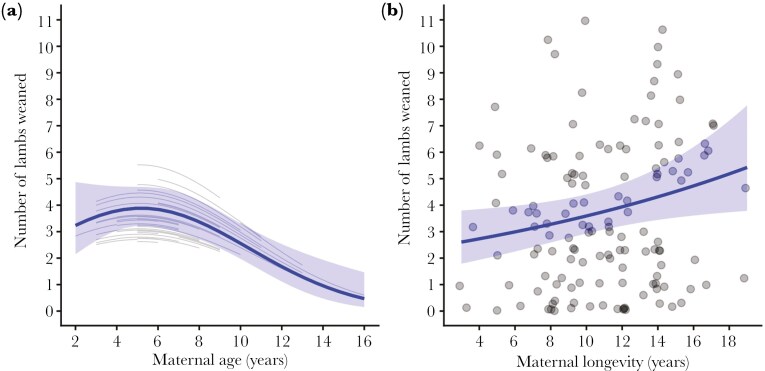
Female lifetime reproductive success for bighorn sheep at Ram Mountain, Alberta, Canada (1971 to 2024). Solid lines represent model predictions for female offspring under average female density conditions (male offspring are reported in Table 1). Shaded bands represent 95% confidence intervals while gray lines are modeled trends for individual mothers. (A) Female lifetime reproductive success, measured as the number of lambs weaned, as a function of maternal age at birth. (B) Female lifetime reproductive success as a function of maternal longevity, as estimated by the age at last sighting.

We found evidence of a negative within-mother effect of maternal age on female offspring LRS, indicating a decline in reproductive performance with maternal age, consistent with reproductive senescence (*β*_*W*_* =* -0.14 [-0.27, -0.02], Fig. 2, Table 1). After controlling for within-mother variation in age at reproduction, maternal longevity positively affected the number of lambs weaned by female offspring (*β*_*S*_ = 0.17 [0.03, 0.32], Table 1). However, the small values of the marginal and conditional *R*^*2*^ indicated that fixed and random effects of maternal identity and death year explained little variance in female LRS. The negative effect of maternal age was also evident when LRS was measured as the number of lambs produced (Table S2).

#### Paternal age effects:

We detected no significant effect of paternal age (linear or quadratic) or longevity on female offspring LRS (Table 2, Figure S5). The low marginal *R*^*2*^ (0.12) suggested that little variance was explained by fixed effects.

## Discussion

We observed no influence of parental age on offspring mass at age three for either sex but found a significant negative association between maternal age and offspring longevity and female lifetime reproductive success. There was no effect of paternal age on offspring longevity. Offspring born to mothers with a long lifespan exhibited a longevity advantage compared to those born to mothers with a short lifespan.

Our long-term study of a natural population provided evidence that maternal age and longevity affect postweaning offspring longevity in a long-lived mammal. The quadratic effect indicates that the decline in offspring traits with increasing maternal age becomes more pronounced at older maternal ages, potentially reflecting increasing physiological costs of reproduction or declining maternal investment with age. When we accounted for maternal longevity, the effect of maternal age remained significant, and was slightly stronger. Therefore, maternal lifespan contributes to, but does not fully explain, the relationship between maternal age and offspring longevity. The fact that similar estimates were obtained in both the full and reduced models indicates that the observed maternal age effect is robust and not merely a byproduct of selective disappearance—where only longer-lived mothers contribute offspring at older ages. Maternal longevity is affected by adult mass (Bérubé et al. 1999) and is likely correlated with maternal reproductive potential. The negative effect of maternal age on offspring longevity, therefore, would be partially hidden by a cross-sectional analysis. The negative association between maternal age and offspring longevity was substantial. Longevity of daughters declined by just over 2 yr as maternal age increased from 3 to 13 yr. A decline in longevity with increasing maternal age was also obvious for sons (Table 1). Paternal age did not appear to affect offspring longevity. Very few studies of wild animals have been able to monitor the effects of paternal age on offspring fitness (Bouwhuis et al. 2015; Fay et al. 2016). Because not all paternities were known, and males have a shorter life expectancy than females (Loison et al. 1999), our results were affected by smaller sample size compared to that available for mothers. Our observations suggest a potential link between paternal age and offspring longevity, warranting further investigation.

Maternal longevity emerged as a critical factor affecting offspring longevity, supporting earlier work that indicated a nonrandom distribution of longevity in bighorn ewes (Bérubé et al. 1999; Festa-Bianchet and King 2007). Longevity is not significantly inheritable in this population (Coltman et al. 2005; Poissant et al. 2008), therefore this effect is likely driven by environmental factors that affect reproductive potential. Ewes that survive to old age are heavier as young adults (Bérubé et al. 1999) and likely have higher reproductive potential at all ages. We observed a clear trend for mothers with longer lifespan to have daughters and sons with extended longevity, while mothers that die young tend to produce offspring of lower life expectancy (Fig. 1).

After accounting for maternal longevity, the negative effect of maternal age on offspring longevity became more evident. Because offspring born to longer-lived mothers exhibit a significant advantage in longevity compared to those born to mothers with shorter lifespan, ignoring maternal longevity underestimates the effect of maternal age on offspring longevity. Given that mothers that die young can only contribute offspring in the first few years of possible maternal ages, if longevity is ignored short-lived mothers would appear to weaken the relationship between maternal age and offspring longevity. This result highlights the importance of including parental lifespan as a covariate in predictive models to properly understand the effects of parental age on offspring traits. By accounting for longevity, our analyses reveal that offspring born to a given mother when she is older have a lower life expectancy than offspring born to that same mother when she is younger (van de Pol and Verhulst 2006).

Consistent with other studies (Schroeder et al. 2015; Reichert et al. 2020), we also detected an effect of maternal age on offspring lifetime reproductive success (Fig. 2). Longevity is a major determinant of lifetime reproductive success in bighorn sheep females, that can only produce one lamb per year. Therefore, given the negative effect of maternal age on offspring longevity, it is not surprising that we also found a negative effect of maternal age on daughter reproductive success. As expected, offspring longevity in relation to maternal age followed a bell-shaped trend and declined more rapidly as mothers aged. This result supports earlier work indicating a slight increase in lamb survival to one year as ewes progressed from ages 2 to 3 to prime-aged (4 to 12 yr), followed by reproductive senescence at about 13 yr. Reproductive senescence, however, only seemed to affect lamb production, not lamb survival to one year (Festa-Bianchet and King 2007). It is important to note, though, that only a few ewes survive to 13 yr of age or older. Lamb mass has a positive effect on survival to one year (Festa-Bianchet et al. 1997), therefore individuals of very poor phenotype as lambs were unlikely to enter our sample.

The decreased longevity of offspring of both sexes, and decreased lifetime reproductive success of females born to older mothers, suggest that older mothers do not provide as much maternal care as prime-aged mothers, and that this lower level of care manifests itself well after the period of maternal care. Research on both bighorn sheep (Martin and Festa-Bianchet 2011) and other ungulates (Morin et al. 2016) suggests that older mothers adopt a more conservative maternal allocation tactic than younger mothers. It is likely, therefore, that our sample of older mothers with data on offspring fitness after the period of maternal care was partly influenced by reproductive decisions early in the maternal allocation period, so that older mothers in very poor condition were unlikely to produce offspring that survived to one year of age. The negative effects of maternal age suggest that older mothers whose lambs survived to one year of age also reduced maternal allocation compared to when they were younger.

Paternal age had a non significant effect on offspring longevity, and no effect on adult mass of either sex or on lifetime reproductive success of daughters. Therefore, our analyses do not support a Lansing effect for males. This result is relevant to a consideration of the possible effects of trophy hunting on population dynamics (Coltman et al. 2005). Trophy hunting substantially decreases the average age of males in a population (Schindler et al. 2017) and likely reduces the average age of fathers. Our analyses do not suggest any strong effects of paternal age on offspring fitness post weaning. Because of the high natural mortality of adult males (Loison et al. 1999) and the additional mortality from trophy hunting up to 1996 (Pigeon et al. 2016), our sample of fathers older than 8 yr was small.

The small sample available for fathers older than about 8 yr and for mothers older than about 13 yr is a limitation of our study, reducing the statistical power to detect any strong senescence-related effects. For males, it would be interesting to study an unhunted population with more fathers aged 9 yr and older. For females, however, because so few individuals survive to age 13 or older (Loison et al. 1999), any effects of very old females on population dynamics are likely to be limited.

By analyzing a unique long-term database on marked known-age individuals monitored through their lifetime, our research provided valuable insights on the effects of parental age on offspring fitness, and therefore on population dynamics. These findings improve our understanding of evolutionary ecology and population dynamics of long-lived mammals. They are also relevant for wildlife management and conservation efforts. By unraveling the interplay between parental age, sex, longevity, and offspring traits, our study underscores the need for sustained, long-term monitoring of natural populations. The challenges posed by small sample sizes, especially in assessing paternal age effects, emphasize the necessity of continuing research to better understand the complexities of population dynamics. Based on our findings, future studies exploring the impact of parental age on offspring life-history evolution should account for parental longevity and investigate sex-specific effects in both parents and offspring. Our results support the increasing evidence (Priest et al. 2002; Bouwhuis et al. 2015; Fay et al. 2016; Hellmann et al. 2020; Monaghan et al. 2020) that parental age can play a role on offspring fitness and that the age structure of parents may affect long-term population dynamics.

## Supplementary Material

araf046_suppl_Supplementary_Materials

## Data Availability

Analyses reported in this article can be reproduced using the data provided by Marchand et al. (2025).
