# Supplementary material for: A family history: effects of parental age on offspring life-history traits in bighorn sheep
Source: Behav Ecol. 2025 May 8;36(4):araf046. doi: 10.1093/beheco/araf046 (PMC12166657; doi:10.1093/beheco/araf046)
Supplement: araf046_suppl_Supplementary_Materials [file araf046_suppl_supplementary_materials.docx]

*Title:*

A family history: Effects of parental age on offspring life-history traits in bighorn sheep

*Authors:*

Emmanuelle Marchand^1*^, Limoilou-Amélie Renaud^2^ and Marco Festa-Bianchet^1^

*Institutional affiliations:*

^1^ Département de biologie, Université de Sherbrooke, 2500, boul. de l'Université, Sherbrooke, Québec, J1K 2R1, Canada

^2^ École d’études autochtones, Université du Québec en Abitibi-Témiscamingue, Québec, J9X 5E4, Canada

**e-mail address of the author responsible for receiving correspondence:*

emmanuelle_marchand0802@hotmail.com

*Short title:*

Parental age affects offspring longevity

Supplementary Information

Table S 1 Summary of descriptive statistics for 978 bighorn sheep offspring from 52 cohorts at Ram Mountain, Alberta, Canada, 1971 to 2024. The correlations between female offspring traits were 0.87 (lifetime reproductive success – longevity), 0.02 (mass at three years – longevity), and 0.12 (lifetime reproductive success – mass at three years). In males, the correlation between longevity and mass at three years was -0.16; reproductive success was not analyzed for males.

| Variable | Description | Minimum | Maximum | Mean | Median | Sample size |
| --- | --- | --- | --- | --- | --- | --- |
| Female offspring mass at three years | Mass at three years, adjusted to September 15 | 51.1 | 78.8 | 64.5 | 64.5 | 230 |
| Male offspring mass at three years |  | 64.8 | 106.0 | 83.6 | 83.8 | 123 |
| Female offspring longevity | Lifespan, estimated by the age at last sighting | 1 | 19 | 6.2 | 6 | 319 |
| Male offspring longevity |  | 1 | 14 | 4.4 | 4 | 268 |
| Female offspring lifetime reproductive success | Number of weaned lambs (unless specified otherwise in text). | 0 | 11 | 3.2 | 2 | 242 |
| Age mother | Maternal age at birth | 2 | 17 | 6.6 | 6.0 | 284 |
| Age father | Paternal age at conception | 2 | 14 | 6.4 | 6.0 | 93 |
| Longevity mother | Maternal longevity | 2 | 19 | 10.4 | 10.0 | 284 |
| Longevity father | Paternal longevity | 2 | 14 | 8.6 | 9.0 | 93 |
| Density | Population density of females (2 years and older) at offspring birth | 16 | 103 | 52.8 | 47.0 | 52 (cohorts) |

Table S 2 Estimates of fixed effects and standard deviations of random intercepts, along with their corresponding 95% confidence intervals (95% CI), from models examining the relationship between female lifetime reproductive success, estimated by the total number of lambs produced, and maternal age in bighorn sheep at Ram Mountain, Alberta, Canada (1971 to 2024). Estimates are presented on the log scale. Maternal identity and death year explained negligible amounts of variance.

| Parameter | Estimate | SE | 95% CI |
| --- | --- | --- | --- |
| Intercept | 1.65 | 0.10 | 1.44, 1.83 |
| Maternal age | -0.16 | 0.07 | -0.30, -0.01 |
| Maternal age^2^ | -0.06 | 0.06 | -0.17, 0.05 |
| Female density at birth | 0.00 | 0.08 | -0.16, 0.16 |
| Maternal longevity | 0.14 | 0.07 | 0, 0.28 |


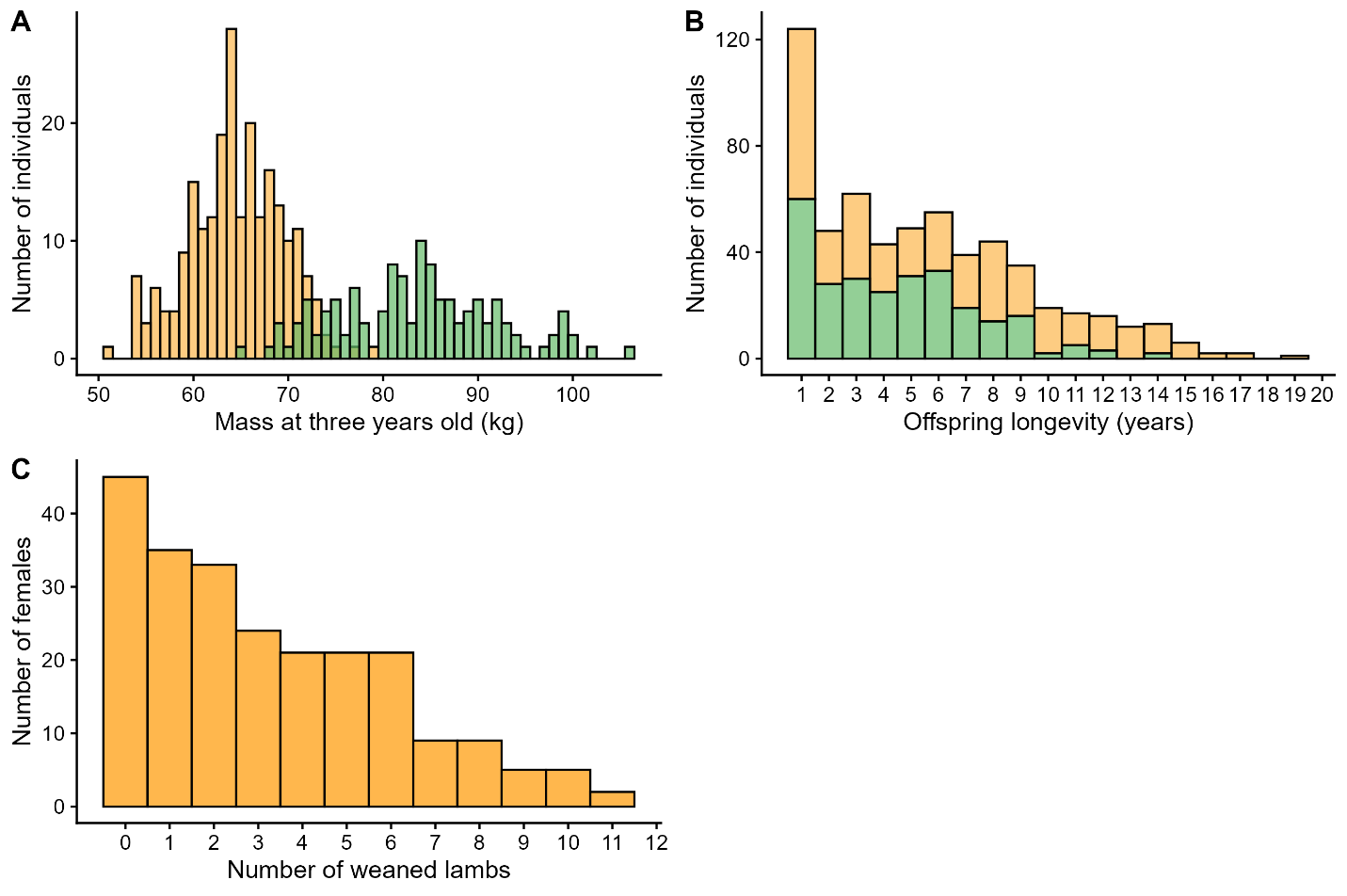


Figure S 1 Frequency distributions of three age-dependant reproductive traits in bighorn sheep at Ram Mountain, Alberta, Canada (1971 to 2024). (A) Offspring mass at three years old, (B) offspring longevity, as estimated by the age at last sighting, (C) offspring lifetime reproductive success, as measured by the number of weaned lambs (for females only). Panels A and C include only individuals one year and older. Female offspring appear in orange, male offspring in green.


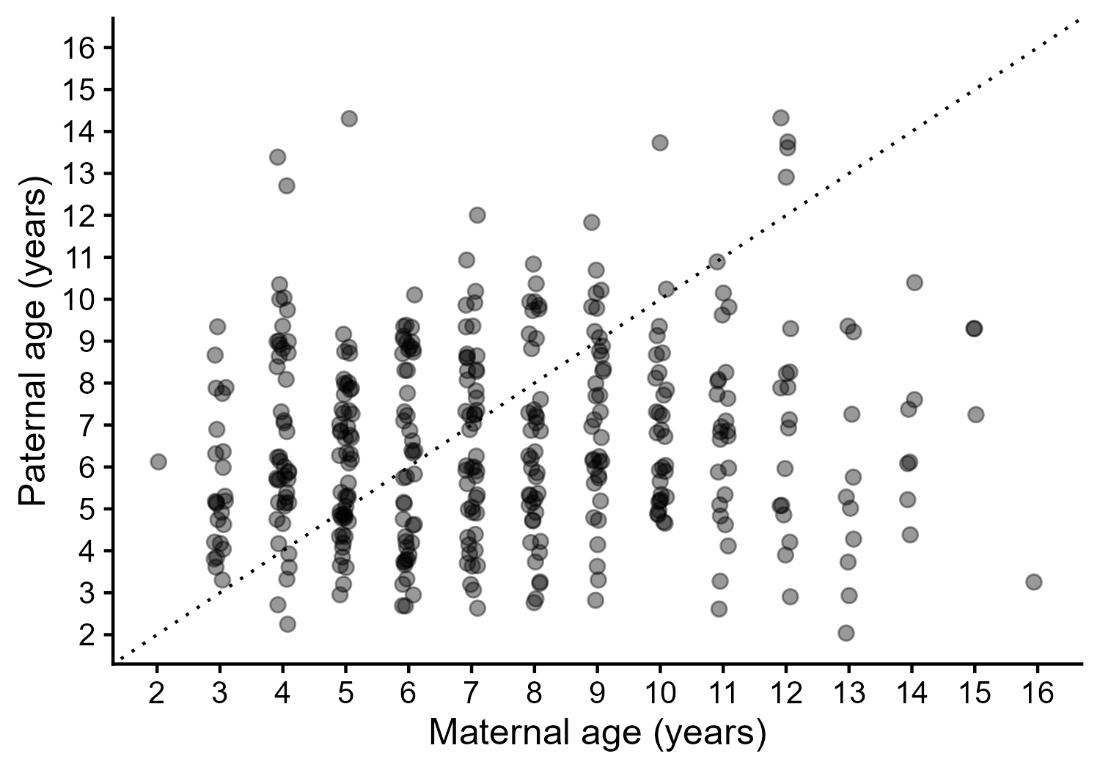


Figure S 2 Relationship between maternal and paternal age for individual offspring in bighorn sheep, Ram Mountain, Alberta, Canada (1988 to 2024). Each point represents a single offspring, with maternal age (x-axis) measured as age at birth and paternal age (y-axis) measured at the time of conception (siring). The Pearson correlation between maternal and paternal age is 0.10 [95% CI: 0.01, 0.20]. The dotted line represents the 1:1 relationship. Data points are slightly jittered for visualization

**
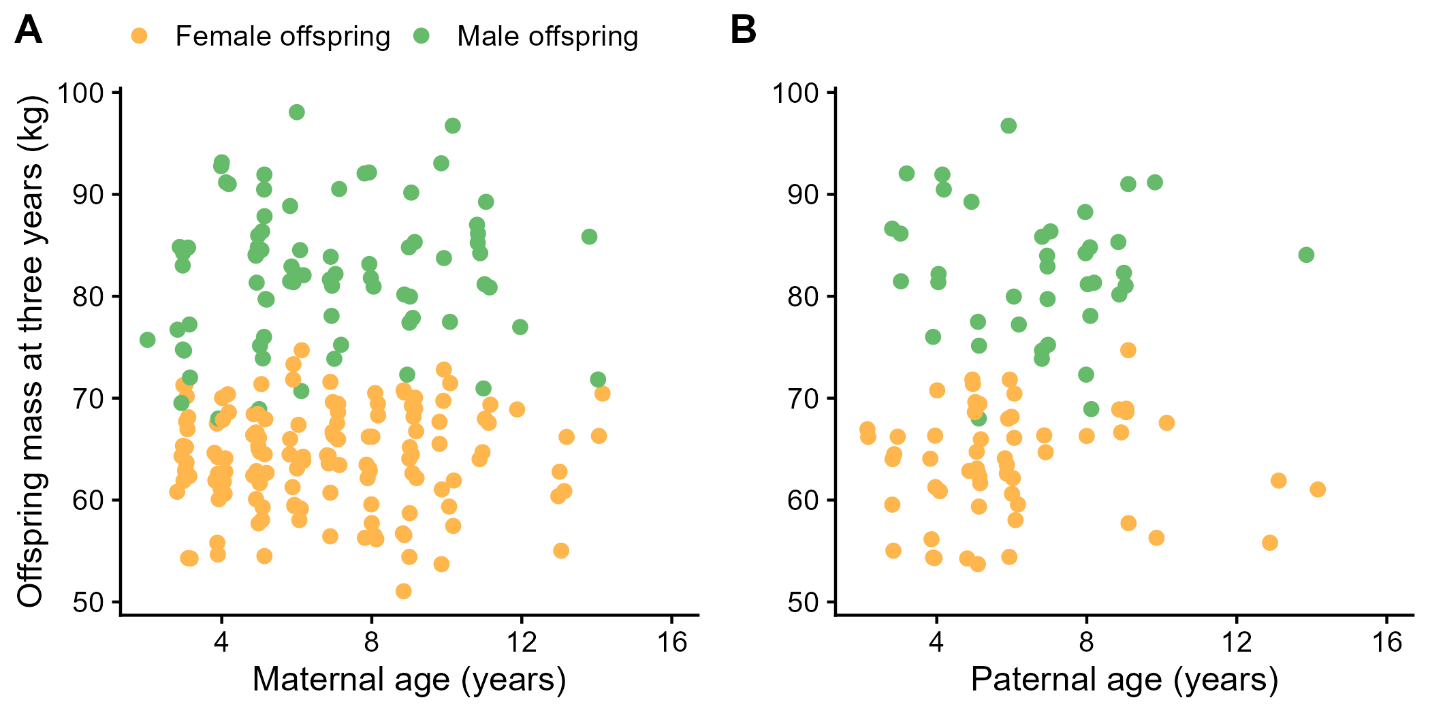
**

Figure S 3 Offspring mass at three years of age in bighorn sheep from Ram Mountain, Alberta, Canada (1971 to 2024). Mass was adjusted to September 15 to account for variation in capture dates. (A) Offspring mass at three years in relation to maternal age at birth. (B) Offspring mass at three years in relation to paternal age at conception. Data points are slightly jittered for visualization. No significant relationships were detected.


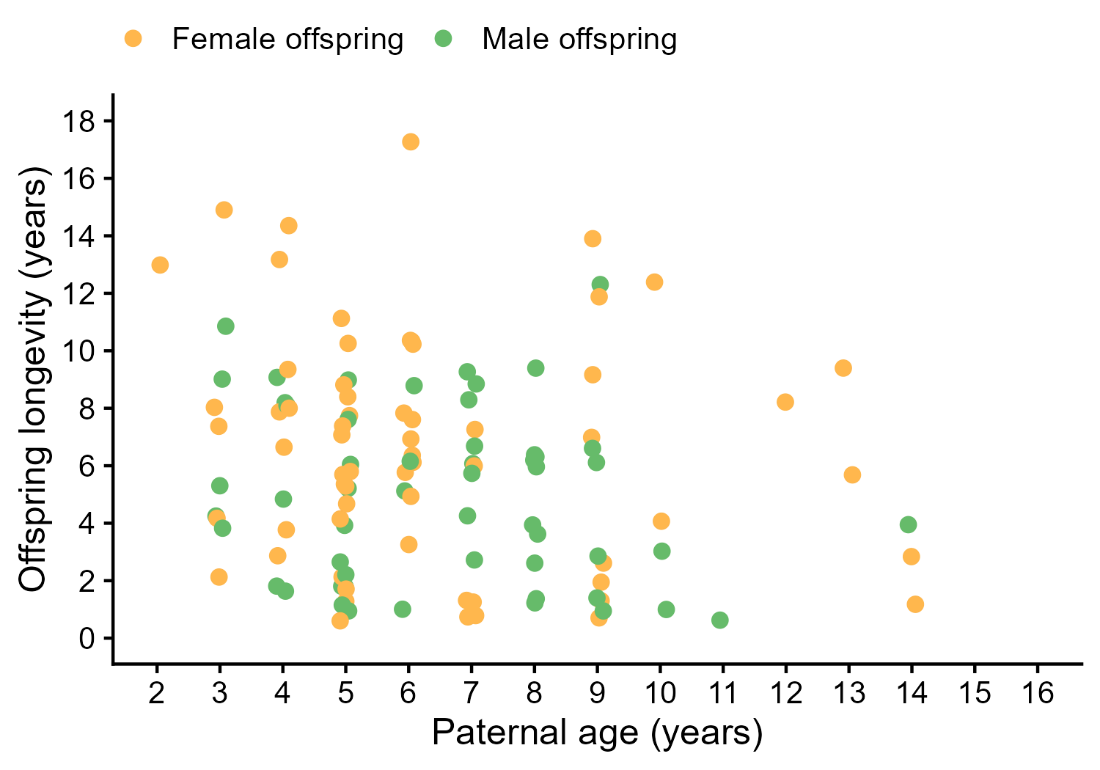


Figure S 4 Offspring longevity in relation to paternal age at conception in bighorn sheep at Ram Mountain, Alberta, Canada (1988 to 2013). Data points are slightly jittered for visualization. No significant relationships were detected.

**
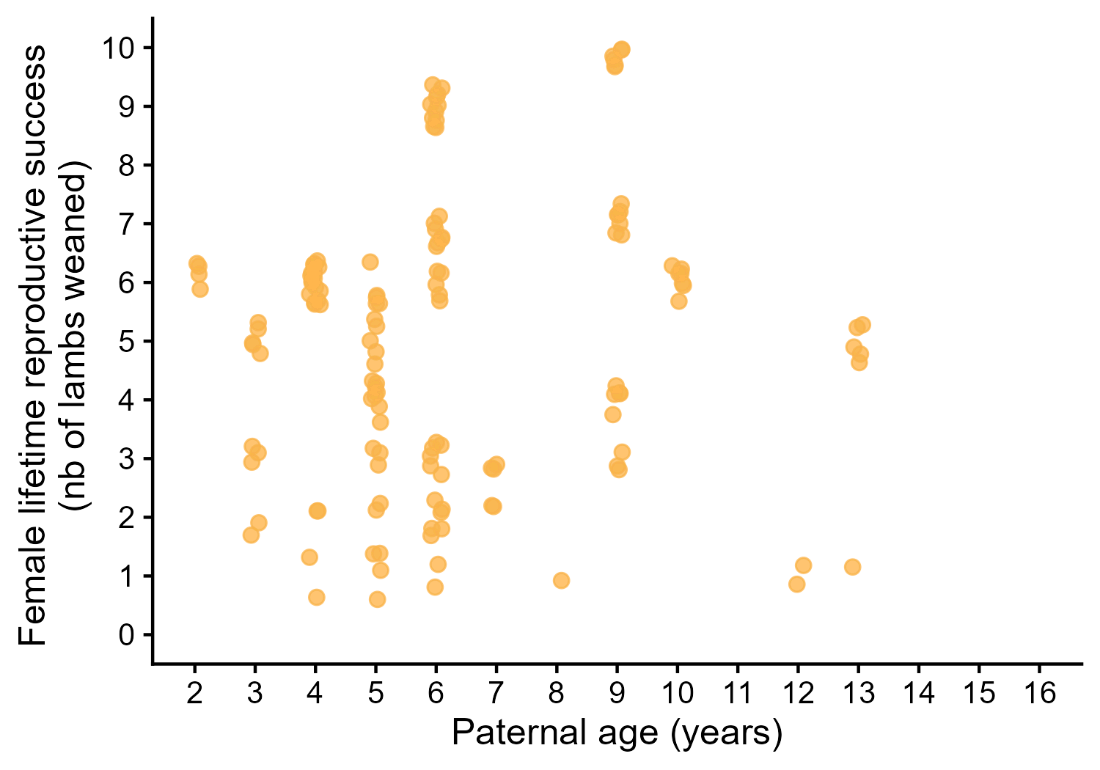
**

Figure S 5 Female offspring lifetime reproductive success in relation to paternal age at conception in bighorn sheep at Ram Mountain, Alberta, Canada (1988 to 2024). Data points are slightly jittered for visualization. No significant relationships were detected. Lifetime reproductive success is the number of lambs that survived to weaning (September).
